# *Drosophila melanogaster* sperm turn more oxidative in the female

**DOI:** 10.1242/jeb.247775

**Published:** 2024-08-07

**Authors:** Cornelia Wetzker, Christin Froschauer, Christian Massino, Klaus Reinhardt

**Affiliations:** ^1^ Applied Zoology, Faculty of Biology, Technische Universität Dresden, 01062 Dresden, Germany; ^2^ B CUBE - Center for Molecular Bioengineering, Technische Universität Dresden, 01307 Dresden, Germany

**Keywords:** FLIM, NADH, FAD, Sperm, *Drosophila*, Metabolism

## Abstract

Males and females of many species store sperm for extended periods. During storage, sperm are predicted to undergo cellular and functional changes, especially towards glycolytic energy metabolism because oxygen radicals derived from oxidative phosphorylation can affect sperm motility and fertilisation ability. However, not all species can use both major energy metabolism pathways. Here, we examined the fruit fly *Drosophila melanogaster* and asked whether sperm metabolism can be fuelled by both glycolysis and oxidative phosphorylation, and to what extent metabolism changes during storage. Inhibiting glycolysis *in vitro* led to a more oxidative state of sperm; inhibiting oxidative phosphorylation increased the glycolytic component, assessed by multi-photon autofluorescence lifetime imaging (FLIM) of NAD(P)H. We further examined sperm in male and female sperm storage organs using FLIM of NAD(P)H and FAD. In intact storage organs, we found that, unexpectedly, (i) sperm were more oxidative in females than in males, and (ii) oxidative phosphorylation increased with storage duration in females. Our observation that the relative contribution of the two major energy metabolic pathways in *D. melanogaster* sperm differs in males and females and over storage time has important evolutionary implications.

## Teaser

**Summary:** To avoid oxidative damage, fruit fly sperm should produce energy by glycolysis once in the female sperm store; however, we show that sperm employ the alternative energy metabolic pathway of oxidative phosphorylation.

## INTRODUCTION

In all species, sperm cells pass through various environments from manufacture to fertilisation. Often, this passage results in an alteration of sperm parameters (Reinhardt et al., 2015). Males of all species store sperm for some time before mating, and females of internally fertilising species also store sperm before fertilisation (Orr and Brennan, 2015). Therefore, the storage organs in males and females are critical environments for a sperm cell. Neither male nor female sperm storage is particularly well understood in any species, including humans and fruit flies (Orr and Brennan, 2015). However, it is clear that sperm physiology and functionality dramatically change after transfer to the female, collectively subsumed as post-ejaculatory modifications of sperm (PEMS) (Pitnick et al., 2020). Insects are particularly interesting examples to study PEMS because females store sperm for extended periods that can range from weeks in grasshoppers, dragonflies, bedbugs or fruit flies (e.g. Ridley, 1988; Reinhardt et al., 1999; Reinhardt, 2005; Reinhardt and Ribou, 2013, Tan et al., 2013) to years in honey bees (Koeniger, 1986), and decades in some ant species (Keller, 1998).

The female storage organ provides a highly complex environment for sperm cells. First, the secretions from the female reproductive tract contain a highly diverse set of molecules that affect sperm functions and their regulatory processes (e.g. Findlay et al., 2008; Prokupek et al., 2009; Schnakenberg et al., 2011; reviewed in LaFlamme and Wolfner, 2013; Wigby et al., 2020). Second, the female storage organ represents highly specific physical conditions such as hypoxia or anoxia, and high pH (Paynter et al., 2017; Gotoh et al., 2023). Third, it is often only in the female reproductive tract and in the storage organs that sperm are exposed to the seminal fluid. The seminal fluid itself contains many different proteins, sugars, lipids, RNA and other molecules (Poiani, 2006, Avila et al., 2011). These molecules affect both sperm and female physiology to alter sperm storage (Avila et al., 2011).

Sperm cells have a very small cytoplasm and, therefore, few energy resources. To save energy, and to avoid sperm ageing, sperm have reduced, or no, motility or reduced energy expenditure in the female sperm store (Reinhardt, 2007; Pitnick et al., 2020). The fact that sperm do not form a tissue in the female storage organ has stimulated hypotheses about how sperm stay alive outside the body of production for a long time. Broadly, they fall into three categories. First, females are widely suggested to nourish sperm (examples in Heifetz and Rivlin, 2010). This idea accepts that living cells must be supplied with resources but neglects that caloric restriction is the most important mechanism overall to prolong cellular lifespan. A second set of hypotheses suggests that females are selected to make the sperm environment as benign as possible and so remove imbalances of sperm homeostasis (Heifetz and Rivlin, 2010). This strategy would involve the increased expression of antioxidants, or antibacterial activity or heightened water balance (Heifetz and Rivlin, 2010; Collins et al., 2006; Dávila et al., 2018). A third group of hypotheses proposes that females suppress sperm metabolism, or certain pathways of it, to reduce sperm metabolic activity, thereby saving endogenous energy substrates, preventing water loss or reducing the production of metabolic oxygen radicals (Ribou and Reinhardt, 2012; Reinhardt and Ribou, 2013; Paynter et al., 2017; Gotoh et al., 2023). Here, we used metabolic measurements to focus on this last aspect but are able to contribute relevant information to the other two hypotheses.

Previous research to address hypotheses about the mechanism of sperm storage has encountered several problems. One problem is that some known alterations during female storage, such as reduced sugar concentrations, pH changes (Koeniger, 1986; Gotoh et al., 2023) or altered enzyme concentrations (Rangel et al., 2021; Paynter et al., 2017) may not be assigned with certainty to one of these hypotheses. A second problem is the lack of an adequate treatment control. For example, honeybee sperm survive in the female for several months. However, sperm extracted from the male also stayed alive for months when stored at room temperature on a laboratory bench (Collins, 2000). A third problem is that sperm metabolism is largely studied *in vitro*. This has the advantage that individual male or female factors or experimental conditions can be isolated (Lahnsteiner, 2014; Paynter et al., 2017; Alves et al., 2021; Massino et al., 2022; Gotoh et al., 2023). However, it has the disadvantage that the ambient oxygen conditions in the laboratory are likely to trigger aerobic metabolism – an unnatural situation, given that in the female sperm storage organs low-oxygen conditions prevail (Paynter et al., 2017; Gotoh et al., 2023).

Several recent studies (Wetzker and Reinhardt, 2019; Turnell and Reinhardt, 2020; Turnell et al., 2021a,b; Turnell and Reinhardt, 2022) employed fluorescence lifetime imaging microscopy (FLIM), a label-free and sensitive approach to assess sperm metabolic parameters in intact, dissected tissues termed *ex vivo*, or in whole organisms. With this method, the biochemical states of the metabolic coenzymes nicotinamide adenine dinucleotide in reduced form (NADH) and flavin adenine dinucleotide (FAD) based on their autofluorescence decay upon laser excitation, being sensitive to their immediate physiochemical environment (Forster, 1976, Clegg et al., 2003; Becker, 2012). As NADH and its phosphorylated form NADPH are spectrally indistinguishable, the notation NAD(P)H addresses both cofactors. Under most circumstances, the concentration of NADPH in comparison with that of NADH is very low, so that the majority of NAD(P)H autofluorescence signal originates from NADH. The method measures overall fluorescence decay times of NAD(P)H and FAD, called mean lifetime, but also short and long lifetimes in the case of several conformational states of the coenzymes that affect the lifetime. The free form of NAD(P)H, which is prominent in glycolysis, has lifetimes in the range of 0.4 to 0.5 nanoseconds (ns), called the short NAD(P)H lifetime. The protein-bound form, prominent in oxidative phosphorylation (OXPHOS), exhibits longer and enzyme-dependent lifetimes in the range of 2 to 6 ns (Iweibo, 1976; Lakowicz et al., 1992; Stringari et al., 2012), called the long NAD(P)H lifetime. Thus, the mean NAD(P)H lifetime as well as the relative proportion of the short lifetime component are proxies for the glycolytic and oxidative states of cells and tissues. Compared with glycolysis, OXPHOS requires an aerobic environment and yields more ATP molecules. The decision of the balance of metabolic pathways is critical for energetic supply but strongly dependent on energetic needs and the tissue environment.

For FAD, the short lifetime fraction reflects protein-bound FAD, ranging from 0.04 to 0.65 ns, while the free FAD produces longer lifetime fractions of 1.7 to 3.2 ns (Chance et al., 1979; Nakashima et al., 1980; Skala et al., 2007; Meleshina et al., 2017). Using FLIM, *Drosophila* sperm proved highly glycolytic *ex vivo* in their intact tissue environment (Wetzker and Reinhardt, 2019) but the contribution of oxidative biochemical pathways was not experimentally tested.

Previous studies found that oxygen radicals are produced in *Drosophila* sperm (Guo et al., 2020; Turnell and Reinhardt, 2020) *in vitro*, indicating the presence of oxygen. We, therefore, expected that *Drosophila melanogaster* sperm are able to use both energy pathways. We confirmed this prediction by separately inhibiting glycolysis and OXPHOS *in vitro*. The ability of sperm to use both pathways then led us to examine the relative proportion of glycolysis and OXPHOS in the female reproductive tract. We expected that sperm would be more glycolytic in the female reproductive tract, thereby avoiding the production of oxygen radicals by OXPHOS. This expectation led to the prediction that (the more glycolytic) sperm in the female would show a larger proportion of free NAD(P)H that contributes to the fluorescence lifetime. We also predicted that over the duration of storage in females, the proportion of free NAD(P)H would increase.

## MATERIALS AND METHODS

### Fly maintenance, sperm dissections and chemical treatment

Wild-type isogenic *D. melanogaster*, natively derived from Dahomey, West Africa, today Benin, were employed for *in vitro* metabolic inhibition measurements, and animals of the strain Canton S were used for *ex vivo* measurements in storage organs. Flies were maintained on standard corn meal molasses yeast agar medium with 90 g l^−1^ corn meal, 40 g l^−1^ yeast, 100 g l^−1^ sucrose, 12 g l^−1^ agar, 40 ml l^−1^ nipagin (10% in ethanol) and 3 ml l^−1^ propionic acid in water at 25°C in an incubator with a 12 h:12 h light:dark cycle.

For *in vitro* metabolic inhibition, male offspring were isolated within hours after eclosion and collected in separate food vials in groups of 20. For dissections, male flies aged 5–6 days were placed in 70% ethanol and then washed in PBS for 30 s each. The reproductive tracts were dissected in PBS with fine forceps using a binocular microscope. Seminal vesicles were then disconnected and transferred individually to a 40 µl drop of treatment or control solution containing fructose and/or chemical inhibitors in the respective cases for each measurement. The solutions contained PBS with metabolic inhibition by 24 mmol l^−1^ 2-deoxy-d-glucose (2DG; D8375, Sigma-Aldrich, Hamburg, Germany, CHEBI:15866) or the combination of 10 µmol l^−1^ rotenone (R8875, Sigma-Aldrich, CHEBI:28201) and 10 µmol l^−1^ antimycin A (A8674, Sigma-Aldrich, CHEBI:2762) or a PBS control. Seminal vesicles were carefully opened with fine needles to release the sperm mass into the solution and incubation timing started. Samples, covered with a clay-footed coverslip and sealed with nail polish, were treated for 15 min prior to FLIM measurements.

2DG is a competitive glucose analogue and, in its phosphorylated state, blocks glycolysis in the cytoplasm (Woodward and Cramer, 1952; McComb and Yushok, 1964). Rotenone and antimycin A are inhibitors of the electron transport chain in the inner mitochondrial membrane, thus OXPHOS, through additive blockade of protein complexes. Rotenone inhibits complex I, NADH dehydrogenase (Chance and Hollunger, 1963; Machinist and Singer, 1965). Antimycin A inhibits complex III, essential for reduction of oxygen to water (Keilin and Hartree, 1955).

The *ex vivo* study was performed blind to treatment. Sample preparation was carried out by a different investigator to that for imaging. Animals and samples were pseudonymised throughout imaging, image processing and quantification of lifetime parameters. Newly emerged virgin male and female flies were collected under CO_2_ anaesthetisation within 6 h post-eclosion and kept in groups of 25. Males and females aged 5 days were set up in pairs for a single timely recorded mating event. Pairs were separated after mating and maintained individually in food vials with transfer to new vials twice per week until the assigned day of sampling. To increase statistical power, data for the female and the male it was mated to were analysed in a paired design (see ‘Data visualisation and statistical analysis’, below).

Fluorescence lifetime imaging was performed within a maximum of 30 min after mating termination (termed 0 days), or 6 h, 1, 2, 3, 7, 14 or 30 days post-mating. Female storage organs were excluded from imaging 30 days post-mating because of insufficient sperm numbers. For dissections, flies were anaesthetised for 10 s with CO_2_ and the reproductive organs were dissected with fine forceps in phosphate-buffered saline (PBS) using a binocular microscope. All reproductive tissues were placed in 40 μl PBS, disentangled carefully if required, covered with a clay-footed coverslip and sealed with nail polish prior to imaging. Imaging was performed at a maximum of 10 min post-dissection.

### FLIM measurements

FLIM microscopy on the basis of time-correlated single photon counting (TCSPC) (Becker et al., 2004) was performed using a FLIM-capable upright laser scanning microscope system with two-photon excitation as described earlier (Wetzker and Reinhardt, 2019) using a water immersion objective (LD C-Apochromat 40×/1.1 W, 421867-9970-000, Carl Zeiss, Jena, Germany). The setup includes an upright AxioExaminer.Z1 (Carl Zeiss) with an *xy*-motorised stage and a Chameleon Ultra II two-photon titanium:sapphire laser (tunable range 690–1080 nm, 80 MHz repetition rate, 140 fs pulse width, Coherent, Saxonburg, PA, USA). Sperm samples were localised by transmission light illumination. NAD(P)H was excited with light of 740 nm and band pass-restricted emission in the range 450–30 nm (AHF analysentechnik AG, Tübingen, Germany), FAD was excited with light of 900 nm and emission in the range of 525–39 nm. NAD(P)H and FAD were imaged sequentially and emitted light was split spectrally at 505 nm onto two hybrid GaAsP detectors (HPM-100-40, Becker&Hickl GmbH, Berlin, Germany).

The total photon count rate was limited to a maximum of 8×10^5^ per second by laser power limitation by use of the internal acousto-optical modulator to prevent sample damage and the saturation of the detection system. Images were acquired using SPCM software version 9.77 for *ex vivo* and 9.80 for *in vitro* measurements (Becker&Hickl GmbH) at 512×512 pixels image size with a pixel dwell time between 3 and 5 μs, yielding a total scan time of approximately 120 s. For size estimates, 6 μm beads were imaged. For all samples, the optical plane with the largest surface of lumen filled with sperm cells was chosen for imaging.

### Fluorescence lifetime data analysis, export and visualisation

In all measurements, fluorescence lifetime analysis was performed in a blinded manner through pseudonymisation of image names. SPCImage software version 6.5 was used for *in vitro* measurements and versions 6.5 and 7.3 for *ex vivo* measurements (Becker&Hickl GmbH).

For *in vitro* measurements, fluorescence lifetime data were fitted double-exponentially with a pixel binning of ‘3’ and with non-fixed parameters short lifetime (τ_1_) and long lifetime (τ_2_), a fixed scatter of ‘0’ and fixed shift values for each image. Lifetime data were exported from image areas with sufficient sperm mass, as a whole image or region of interest.

For *ex vivo* measurements, data of images with blinded nomenclature were binned to ‘10’ to identify optimal shift values for double-exponential [NAD(P)H] and triple-exponential (FAD) fluorescence decays. The scatter was fixed to ‘0’. Lifetime values were calculated with fixed shift and binning ‘1’ for NAD(P)H and binning ‘3’ for FAD because of lower photon counts. Mean lifetime (τ_m_) was calculated by addition of the products of τ_1_ and τ_2_ with their respective fractions, a_1_% and a_2_%, for NAD(P)H (τ_m_=τ_1_×a_1_%+τ_2_×a_2_%) by the software. For FAD, three lifetime components, τ_1_, τ_2_ and τ_3_, with their respective fractions, a_1_%, a_2_% and a_3_%, were supported by better χ^2^ values (τ_m_=τ_1_×a_1_%+τ_2_×a_2_%+τ_3_×a_3_%). Images of high magnification were applied to quantitative analyses.

Regions of interest (ROI) that contain sperm in male or female tissues were selected in each color-coded image of mean NAD(P)H and FAD lifetime of low magnification for further analysis (see Fig. S1). For NAD(P)H, clusters representing sperm were selected in phasor plots for further analysis as described earlier to better extract sperm-specific data by background exclusion (Wetzker and Reinhardt, 2019). Lifetime parameters were exported for each image from SPCImage. Exported parameters include the proportions and discrete values of lifetime and the mean lifetime in each ROI, χ^2^ values as indicators of the fit quality, as well as photon counts and pixel numbers to determine the average number of photons for lifetime calculations for each ROI. Quality thresholds, being a minimum of 1500 photons per binned pixel for NAD(P)H and a χ^2^ value below 1.4 as well as a minimum of 1000 photons per binned pixel for FAD, were applied for image selection for further data analysis. Tables that describe detailed information on samples, mating patterns, lifetime fitting, quality thresholds and extracted lifetime parameters for statistical analysis and plotting are available from GitHub (https://github.com/cwetzker/sperm_metFLIM_2024).

### Data visualisation and statistical analysis

Lifetime parameters of images were exported as two-dimensional arrays from SPCImage and read in and plotted using the amber lookup table provided by the cmasher (version 1.6.3) package (van der Velden, 2020) in python (version 3.9) (van Rossum, 1995). All statistics and plots were generated in R (version 4.0.1) using RStudio (version 1.2.5001) and Inkscape (version 1.0.1), except phasor plots exported from SPCImage because of unavailability of raw phasor data. R source code is available from GitHub (https://github.com/cwetzker/sperm_metFLIM_2024).

For *ex vivo* data, variation in mean lifetime and short lifetime fractions of both NAD(P)H and FAD was examined by linear mixed-effects models. We used sperm localisation in male or female (‘location’) and storage duration (‘duration’) as fixed factors. The sperm of a male was investigated both in the male after mating and at one time point in the female. This paired design was analysed by using male ID as a random effect (‘lmer’ of the package ‘lme4’) (Bates et al., 2015). Data were tested for normal distribution using the Shapiro–Wilk test. Type III analysis of variance (ANOVA) tests were used to compare group means.

For *in vitro* data, normal distribution of data was tested using the Shapiro–Wilk test. Variation in short lifetime fractions of NAD(P)H was examined by analysis of variance of aligned rank transformed data (ART ANOVA) (Wobbrock et al., 2011) because of the lack of normal distribution of data, followed by a Tukey's test for comparisons between groups.

## RESULTS

### *Drosophila* sperm employ glycolysis and OXPHOS *in vitro*

We examined sperm samples of male flies and chemically inhibited glycolysis by application of 2DG, and inhibited OXPHOS by a combination of rotenone and antimycin A. If sperm gain ATP through glycolysis, 2DG will decrease the free-to-bound ratio of NAD(P)H, thereby decreasing the percentage contribution of free NAD(P)H to the mean lifetime (a_1_%), and increase the mean NAD(P)H lifetime. If sperm employ OXPHOS, rotenone/antimycin A will increase the free-to-bound ratio of NAD(P)H, decrease NAD(P)H lifetime and increase the free NAD(P)H contribution to the mean lifetime.

**Fig. 1.**
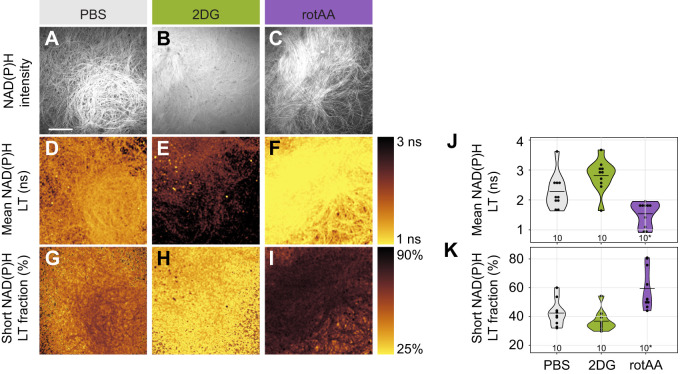
**Mean NAD(P)H lifetime and short NAD(P)H lifetime fraction of *Drosophila melanogaster* sperm *in vitro* upon metabolic manipulation.** (A–I) Example images of NAD(P)H fluorescence intensity (A–C), and pseudo-colour-coded mean NAD(P)H lifetime (LT) (D–F) and short NAD(P)H lifetime fraction (G–I) of dissected sperm are displayed. Cells were exposed to metabolic inhibitors 2-deoxy-d-glucose (2DG) (B,E,H), the combination of rotenone and antimycin A (rotAA) (C,F,I) or phosphate-buffered saline (PBS; control) (A,D,G). Scale bar: 50 µm (applies to all images). (J,K) Violin plots show mean values of NAD(P)H lifetime (J) and short NAD(P)H lifetime fraction (K) across images of individual samples as well as group means with sample sizes per group below. Asterisks indicate groups with non-normal data distribution.

As predicted if sperm use both pathways, the mean NAD(P)H lifetime of sperm was affected by treatment (Fig. 1): it increased to 2.81±0.53 ns after inhibition of glycolysis, compared with PBS controls (2.28±0.58 ns), and decreased to 1.54±0.40 ns after inhibition of OXPHOS by rotenone/antimycin A treatment (Table 1). Sperm density and density×treatment interactions had no significant effect on sperm metabolism (Table S1). Specifically, the percentage that free NAD(P)H contributed to the mean lifetime (a_1_%) (±s.d.) decreased to 36.79±7.15% after glycolysis inhibition, compared with PBS controls (42.41±8.82%), but increased to 59.36±14.37% after inhibition of OXPHOS (Fig. 1).

**Table 1.**
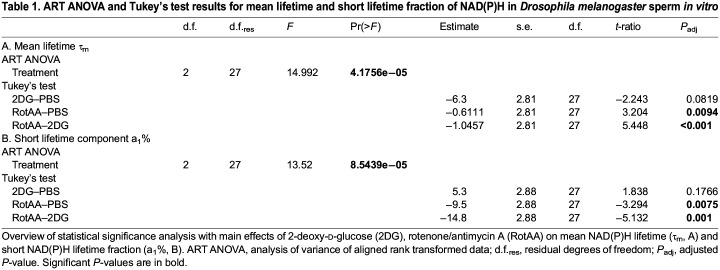
ART ANOVA and Tukey's test results for mean lifetime and short lifetime fraction of NAD(P)H in *Drosophila melanogaster* sperm *in vitro*

Sperm metabolism is likely to be impacted by sperm density. We therefore confirmed that sperm density and interactions of density and treatment had no significant effect on sperm metabolism (Table S2).

### Sperm NAD(P)H lifetime signatures differ between male and female storage organs, and differences increase with extended storage duration

**Fig. 2.**
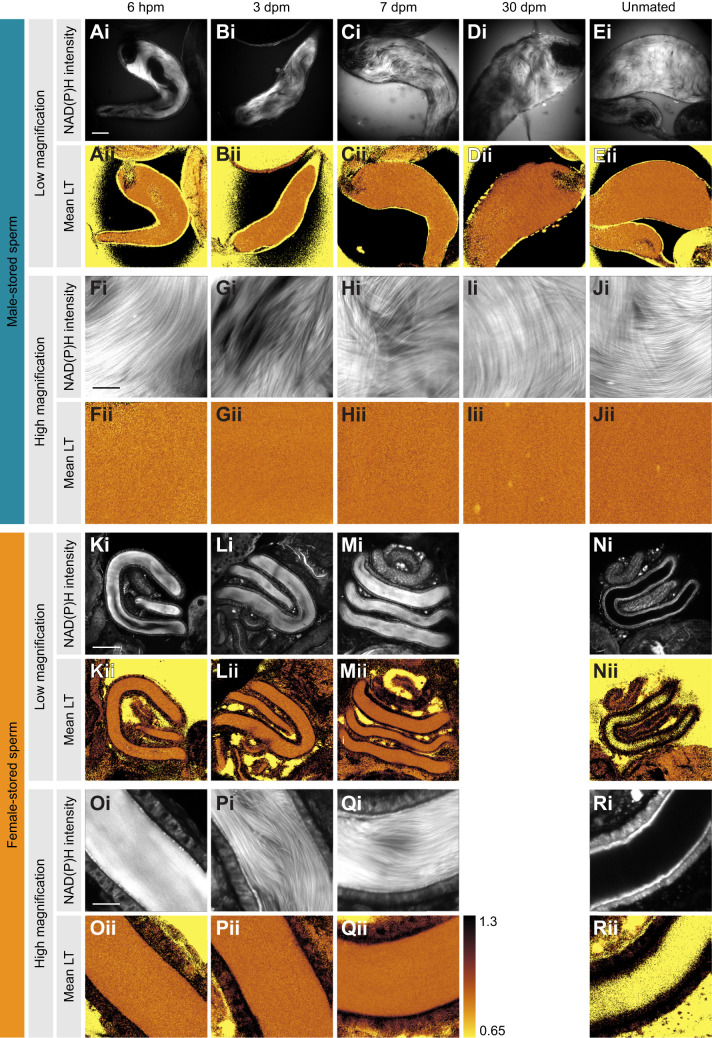
**Sperm metabolism in male and female storage organs over time in *D. melanogaster*.** Example pseudo-colour images of NAD(P)H intensity (Ai–Ji) and mean NAD(P)H lifetime of sperm *ex vivo* in male seminal vesicles (Aii–Jii) and female seminal receptacles (Ki–Ri and Kii–Oii, respectively) at 6 h post-mating (hpm), 3, 7 and 30 days post-mating (dpm) and in unmated controls (aged 7 days), and at low (rows 1 and 2) and high (rows 3 and 4) magnification. Within columns, sperm are from the same male (except for virgin females N and R). Low and high magnification images of each time point are taken from the same biological sample. Unmated males (E and J) and virgin females (N and R) serve as controls. Scale bars: 50 µm (low magnification) and 10 µm (high magnification).

**Fig. 3.**
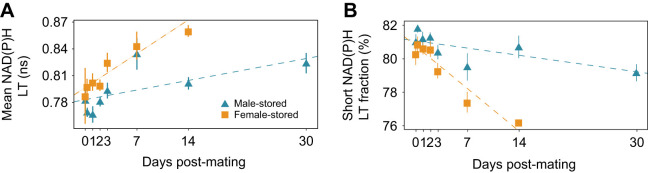
**NAD(P)H lifetime parameters in sperm stored in male and female storage organs for different durations.** Group means (±s.e.m.) of mean NAD(P)H lifetime (A) and short NAD(P)H lifetime fraction (B) of male- and female-stored sperm at time points 0, 1, 2, 3, 7 and 14 days post-mating and at 30 days post-mating for male-stored sperm only.

In both males and females, the sperm NAD(P)H FLIM signal was clearly distinct from the surrounding epithelia (Fig. 2). At every time point representing different storage durations (see Table S1 for sample sizes), the same male's sperm showed slightly longer mean NAD(P)H lifetime in female compared with male storage organs (Figs 2 and 3A, Table 2). Within both males and females, mean lifetime increased with increasing storage duration but much more so in females (Fig. 3A, Table 2; significant interaction effect of storage location and duration). For example, in males, the mean NAD(P)H lifetime (±s.d.) of stored sperm increased from 0.77±0.02 ns 1 day post-mating to 0.82±0.03 ns 30 days post-mating; in females, an increase from 0.80±0.02 ns to 0.86±0.01 ns was observed by 14 days post-mating (Figs 2 and 3A).

**Table 2.**
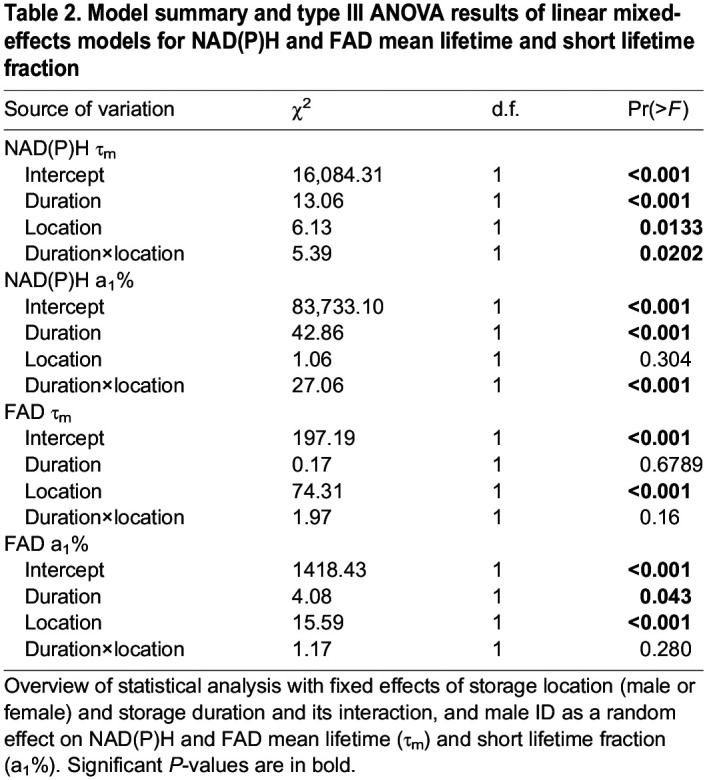
Model summary and type III ANOVA results of linear mixed-effects models for NAD(P)H and FAD mean lifetime and short lifetime fraction

The fraction of short NAD(P)H lifetime (a_1_%) decreased over time in both sexes and again, this was steeper in females than in males (Fig. 3B, Table 2: significant interaction effect of storage location and duration). In detail, free NAD(P)H contributed 81.17±0.99% in male-stored and 80.65±0.97% in female-stored sperm 1 day post-mating, decreasing to 80.71±1.90% in male-stored sperm and 76.22±0.27% in female-stored sperm 14 days post-mating, and further to 79.16% in male-stored sperm 30 days post-mating.

The lifetime increase, but particularly the short lifetime fraction decrease over storage duration indicates that sperm become less glycolytic and more oxidative during sperm storage in females. We further tested whether another parameter of oxidative metabolism, the amount of protein-bound FAD (a_1_%), would be lower in males than in females and increase with storage duration.

### Male- and female-stored sperm differ in FAD lifetime signatures and change over storage time

**Fig. 4.**
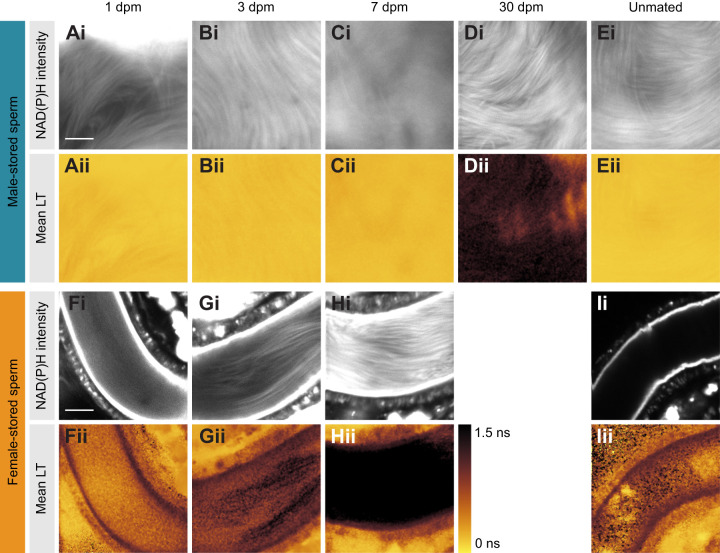
**FAD metabolism in sperm in male and female storage organs over time in *D. melanogaster*.** Example pseudo-colour images of FAD intensity (Ai–Ei) and mean NAD(P)H lifetime of sperm *ex vivo* in male seminal vesicles (Aii–Eii) and female seminal receptacles (Fi–Ii and Fii–Iii, respectively) at 1, 3, 7 and 30 days post-mating (dpm) and in unmated controls (aged 7 days). Scale bar: 10 µm (applies to all images).

**Fig. 5.**
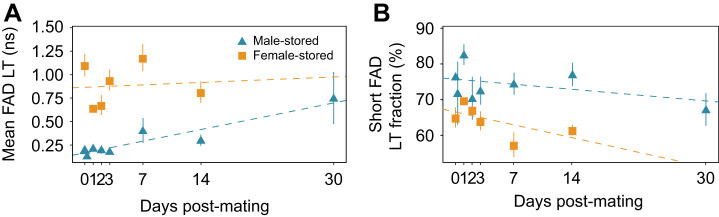
**FAD lifetime parameters in sperm stored in male and female storage organs for different durations.** Group means (±s.e.m.) of mean FAD lifetime (A) and short FAD lifetime fractions (B) of male- and female-stored sperm at time points 0, 1, 2, 3, 7 and 14 days post-mating and at 30 days post-mating for male-stored sperm only.

Pseudo-colour images of mean FAD lifetime of sperm in males and females (Fig. 4) showed shorter mean FAD lifetime of sperm in male storage (Fig. 4Aii–Eii and 5A) than in female storage (Fig. 4Fii–Hii and 5A, Table 2). In males, mean FAD lifetime ranged from 0.22±0.05 ns 1 day post-mating to 0.18±0.07 ns 3 days post-mating, 0.30±0.14 ns 14 days post-mating and 0.75±0.55 ns 30 days post-mating (Figs 4 and 5A). In female storage, mean FAD lifetime of sperm ranged from 0.65±0.04 ns 1 day post-mating, to 0.94±0.27 ns 3 days post-mating and 0.81±0.30 ns 14 days post-mating. The lifetime of sperm of unmated males was identical to that of mated ones (Fig. 4Eii). The increase in mean FAD lifetime over time was not significant (Table 2).

The short FAD lifetime fraction (a_1_%) was higher in male than in female storage. It decreased over time in male storage, from 82.60±5.78% 1 day post-mating to 72.52±10.40% and 77.05±8.09% 3 and 14 days post-mating to reach the lowest value of 67.24±9.16% at 30 days (Fig. 5B). The short FAD lifetime fraction of sperm stored in females decreased from 69.87±1.27% 1 day post-mating to 61.52±4.00% 14 days post-mating (Fig. 5B).

## DISCUSSION

### Metabolic pathways of *Drosophila* sperm and male fertility

We found that inhibition of glycolysis led to a more oxidative state in *D. melanogaster* sperm (Figs 1 and 2, Table 1), indicated by a decrease in the short NAD(P)H lifetime fraction and consequently an increase of the mean lifetime. Inhibiting OXPHOS through simultaneous interference with complexes I and III enhanced the glycolytic state, indicated by an increase in the short NAD(P)H lifetime fraction and a decrease of the mean lifetime. This bidirectional response experimentally supports previous observations (Wetzker and Reinhardt, 2019; Turnell and Reinhardt, 2020) that glycolysis and OXPHOS simultaneously contribute to cellular ATP production in *Drosophila* sperm. In our study, the changes in glycolytic and oxidative state of sperm were relative to each other and not absolute measures. Further, the assay does not quantify ATP production directly. We are, therefore unable to conclude whether the inhibited pathway merely contributed less to ATP production or whether the non-inhibited pathway actively compensated for losses by the other. This would require *ex vivo* or *in vitro* measurements of ATP in sperm and, therefore, manipulations such as use of transgenic animals, and the introduction of transgenic probes, which were not available to us.

The observations that *D. melanogaster* sperm use OXPHOS (Wetzker and Reinhardt, 2019; Turnell and Reinhardt, 2020; Turnell et al., 2021a,b; Turnell and Reinhardt, 2022; this study) show that sperm mitochondria contribute to sperm metabolism. Indeed, mitochondrial OXPHOS effects on sperm motility are known from other species (Tourmente et al., 2015; Piomboni et al., 2012; Moraes and Meyers, 2018). Some studies suggested that the mitochondrial contribution to sperm performance shaped the evolution of competitive male fertility (sperm competition) (Anderson et al., 2005). The exclusive maternal inheritance of mitochondria makes mitochondrial physiology an unlikely candidate upon which selection for high male fertility can operate. However, the number of mitochondria and other mitochondrial genes are under nuclear control and may shape sperm performance. By contrast, genes for glycolytic pathways are inherited from both parents. Sperm competitive ability may, therefore, evolve through males. However, sperm competitive ability, i.e. a male's ability to sire offspring in competition, has very low to no heritability (reviewed in Dobler and Reinhardt, 2016), and experimental data show it to be largely phenotypically plastic (Dobler and Reinhardt, 2016). The notions of a mitochondrial contribution to sperm phenotypic plasticity, and a possible limited evolution of male fertility via mitochondrial traits, deserve more consideration in the sperm competition literature.

### Sperm metabolism differs in male and female storage organs

Our second result was that sperm metabolism differed in males and females. Unexpectedly, from the viewpoint of existing hypotheses, sperm were found to be in a more oxidative state in females than in males, indicated by the decrease in free NAD(P)H. Similar to the other species examined so far – two ant species (Gotoh et al., 2023), the honeybee (Paynter et al., 2017), a cricket (Ribou and Reinhardt, 2012) and the bedbug (Reinhardt and Ribou 2013) – sperm metabolic changes in the females were very rapid, occurring within 30 min of mating (Figs 2 to 5) (Wetzker and Reinhardt, 2019, Ribou and Reinhardt, 2012, Reinhardt and Ribou, 2013). However, compared with those five species, *D. melanogaster* was exceptional in that sperm were more oxidative in the female than in the male. It is not clear whether these differences are related to total sperm storage duration, which in *D. melanogaster* is much shorter than in the other species. In addition, whilst sperm were stored in the female receptacle for 30 days, another part of the sperm is stored in the spermatheca. This organ's accessibility to FLIM is challenging because of the high extent of energy-absorbing molecules such as melanin and we are unable to speculate about the metabolic conditions in the spermatheca.

The major difference in sperm metabolism in males and females was the different temporal variation of its components: the mean NAD(P)H lifetime diverged, FAD a_1_% stayed similar and FAD mean lifetime converged. Although diverging, both NAD(P)H parameters changed in the same direction in males and females, just more strongly in females. This suggests that sperm turn oxidative more rapidly in females than in males. Alternatively, the NAD(P)H sperm metabolism changes similarly in males and females but the continuous sperm production in males causes a constant ‘dilution’ of the stored, more oxidative sperm with the newly manufactured, more glycolytic sperm. In the latter case, the variation, not just the mean, in NAD(P)H parameters is expected to increase with storage time in males (because new cohorts are simply added to existing ones). However, the standard error did not increase over storage time in males (Fig. 3) and we hypothesise that sperm indeed show different metabolism in males and females.

### Sperm metabolism and sperm ageing

Sperm metabolism may be related to a reduction in reproductive fitness caused by sperm ageing in *D. melanogaster*. Although it is unlikely that all sperm ageing effects on reproductive fitness are caused by sperm metabolism, we here discuss some possible connections between them. Offspring resulting from eggs fertilised with sperm that had been in female storage for 15 days had 10–20% lower egg-to-adult viability and reduced egg hatchability; lower larval and lower pupal viability contributed to this effect (Tan et al., 2013). We have not provided offspring data. However, our *ex vivo* data show that NAD(P)H sperm metabolism, but not FAD sperm metabolism, changed over storage time in female *D. melanogaster*, so any metabolic effects on sperm ageing will be related to NAD(P)H not FAD sperm metabolism. If metabolic effects are related to sperm ageing and if we assume our experimental conditions were somewhat comparable to those of Tan et al. (2013), our data would suggest that mean increases of just 70 ps in NAD(P)H mean lifetime, or 5% NAD(P)H a_1_% reductions during female sperm storage, could cause sizable reductions in offspring survival.

A recent comparative study in the genus *Drosophila* suggested that females mate frequently in species whose sperm metabolise highly and oxidatively, possibly to replace sperm that age quickly in an oxidative environment (Turnell and Reinhardt, 2022). At the other end of the spectrum, sperm were largely glycolytic in species where females mated less often. The shape of the sperm ageing curve helps us to predict how males and females would behave to avoid or reduce sperm ageing effects, including when females should re-mate to obtain fresh, undamaged sperm. Our data in *D. melanogaster* suggest that NAD(P)H or FAD metabolic parameters changed linearly while sperm aged in storage. A linear deterioration of sperm quality in the female predicts that females benefit from regular re-mating, rather than re-mating soon after the first mating (if there were a concave sperm ageing curve) or after a long period of storage (for a convex sperm ageing curve). Unfortunately, the shape of the sperm ageing curve in female storage does not seem to be known for other species (Reinhardt, 2007).

### *In vitro* studies of sperm energy metabolism – a cautionary note

A final implication of our results relates to the use of *in vitro* sperm parameters. In honeybees and ants, sperm storage organs were strongly depleted of oxygen (Paynter et al., 2017, Gotoh et al., 2023). In our study, we found that *in vitro*, sperm were clearly much more oxidative than in intact tissue, with absolute values of short NAD(P)H lifetime fractions of 42.41±8.82% in the control group in ambient oxygen conditions, compared with a mean of 80.57±1.46% in intact seminal vesicles. This change indicates a much more oxidative state of sperm *in vitro*, upon release into the oxygen-saturated buffer solutions. It emphasises the importance of the investigation of sperm in an environment as natural as possible to understand the true nature of biochemical processes.

### Conclusion

Our findings demonstrate that both major ATP-producing pathways are used by *D. melanogaster* sperm *in vitro* but also in *ex vivo* natural environments. Our observations underline the importance of the sperm environment for metabolism and consequently sperm functionality. Our unexpected finding of an oxidative sperm metabolism in females begs the question whether *D. melanogaster* females protect the stored sperm against oxygen radicals by producing antioxidants.

## Supplementary Material

10.1242/jexbio.247775_sup1 Supplementary information

## References

[JEB247775C1] Alves, L. Q., Ruivo, R., Valente, R., Fonseca, M. M., Machado, A. M., Plön, S., Monteiro, N., García-Parraga, D., Ruiz-Díaz, S., Sánchez-Calabuig, M. J. et al. (2021). A drastic shift in the energetic landscape of toothed whale sperm cells. *Curr. Biol.* 31, 3648-3655. 10.1016/j.cub.2021.05.062 34171300

[JEB247775C2] Anderson, M. J., Nyholt, J. and Dixson, A. F. (2005). Sperm competition and the evolution of sperm midpiece volume in mammals. *J. Zool. (London)* 267, 135-142. 10.1017/S0952836905007284

[JEB247775C3] Avila, F. W., Sirot, L. K., LaFlamme, B. A., Rubinstein, C. D. and Wolfner, M. F. (2011). Insect seminal fluid proteins: Identification and function. *A. Rev. Entomol.* 56, 21-40. 10.1146/annurev-ento-120709-144823 PMC3925971 20868282

[JEB247775C4] Bates, D., Mächler, M., Bolker, B. and Walker, S. (2015). Fitting linear mixed-effects models using lme4. *J. Stat. Softw.* 67, 1-48. 10.18637/jss.v067.i01

[JEB247775C5] Becker, W. (2012). Fluorescence lifetime imaging−techniques and applications. *J. Microsc.* 247, 119-136. 10.1111/j.1365-2818.2012.03618.x 22621335

[JEB247775C6] Becker, W., Bergmann, A., Hink, M. A., König, K., Benndorf, K. and Biskup, C. (2004). Fluorescence lifetime imaging by time-correlated single-photon counting. *Microsc. Res. Techn.* 63, 58-66. 10.1002/jemt.10421 14677134

[JEB247775C7] Chance, B. and Hollunger, G. (1963). Inhibition of electron and energy transfer in mitochondria. IV. Inhibition of energy-linked diphosphopyridine nucleotide reduction by uncoupling agents. *J. Biol. Chem.* 238, 418-431. 10.1016/S0021-9258(19)84014-0 14019998

[JEB247775C8] Chance, B., Schoener, B., Oshino, R., Itshak, F. and Nakase, Y. (1979). Oxidation-reduction ratio studies of mitochondria in freeze-trapped samples. NADH and flavoprotein fluorescence signals. *J. Biol. Chem.* 254, 4764-4771. 10.1016/S0021-9258(17)30079-0 220260

[JEB247775C9] Clegg, R. M., Holub, O. and Gohlke, C. (2003). Fluorescence lifetime-resolved imaging: measuring lifetimes in an image. *Method. Enzymol.* 360, 509-542. 10.1016/S0076-6879(03)60126-6 12622166

[JEB247775C10] Collins, A. M. (2000). Survival of honey bee (Hymenoptera: Apidae) spermatozoa stored at above-freezing temperatures. *J. Econ. Entomol.* 93, 568-571. 10.1603/0022-0493-93.3.568 10902300

[JEB247775C11] Collins, A. M., Caperna, T. J., Williams, V., Garrett, W. M. and Evans, J. D. (2006). Proteomic analyses of male contributions to honey bee sperm storage and mating. *Insect Mol. Biol.* 15, 541-549. 10.1111/j.1365-2583.2006.00674.x 17069630 PMC1847503

[JEB247775C12] Dávila, F., Botteaux, A., Bauman, D., Chérasse, S. and Aron, S. (2018). Antibacterial activity of male and female sperm-storage organs in ants. *J. Exp. Biol.* 221, jeb175158. 10.1242/jeb.175158 29444845

[JEB247775C13] Dobler, R. and Reinhardt, K. (2016). Heritability, evolvability, phenotypic plasticity and temporal variation in sperm-competition success of *Drosophila melanogaster*. *J. Evol. Biol.* 29, 929-941. 10.1111/jeb.12858 26990919

[JEB247775C14] Findlay, G. D., Yi, X., MacCoss, M. J. and Swanson, W. J. (2008). Proteomics reveals novel Drosophila seminal fluid proteins transferred at mating. *PLoS Biol.* 6, e178. 10.1371/journal.pbio.0060178 18666829 PMC2486302

[JEB247775C15] Forster, L. S. (1976). Fluorescence lifetimes of biomolecules. *Photochem. Photobiol.* 23, 445-448. 10.1111/j.1751-1097.1976.tb07277.x 781696

[JEB247775C16] Gotoh, A., Takeshima, M. and Mizutani, K.-I. (2023). Near-anoxia induces immobilization and sustains viability of sperm stored in ant queens. *Sci. Rep.* 13, 3029. 10.1038/s41598-023-29705-7 36859427 PMC9977914

[JEB247775C17] Guo, R., Henke, A.-L. and Reinhardt, K. (2020). Sperm viability varies with buffer and genotype in *Drosophila melanogaster*. *Fly* 15, 1-7. 10.1080/19336934.2020.1837592 33054517 PMC7808417

[JEB247775C18] Hartley, M., Kleywegt, G. J., Patwardhan, A., Sarkans, U., Swedlow, J. R. and Brazma, A. (2022). The bioimage archive – building a home for life-sciences microscopy data. *J. Mol. Biol.* 434, 167505. 10.1016/j.jmb.2022.167505 35189131

[JEB247775C19] Heifetz, Y. and Rivlin, P. K. (2010). Beyond the mouse model: using *Drosophila* as a model for sperm interaction with the female reproductive tract. *Theriogenology* 73, 723-739. 10.1016/j.theriogenology.2009.11.001 20015541

[JEB247775C20] Iweibo, I. (1976). Protein fluorescence and electronic energy transfer in the determination of molecular dimensions and rotational relaxation times of native and coenzyme-bound horse liver alcohol dehydrogenase. *Biochim. Biophys. Acta* 446, 192-205. 10.1016/0005-2795(76)90110-0 974111

[JEB247775C21] Keilin, D. and Hartree, E. F. (1955). Relationship between certain components of the cytochrome system. *Nature* 176, 200-206. 10.1038/176200b0 13244659

[JEB247775C22] Keller, L. (1998). Queen lifespan and colony characteristics in ants and termites. *Insectes Soc.* 45, 235-246. 10.1007/s000400050084

[JEB247775C23] Koeniger, G. (1986). Reproduction and mating behavior. In *Bee Breeding and Genetics* (ed. T. E. Rinderer), pp. 255-280. New York: Academic Press.

[JEB247775C24] LaFlamme, B. A. and Wolfner, M. F. (2013). Identification and function of proteolysis regulators in seminal fluid. *Mol. Reprod. Dev.* 80, 80-101. 10.1002/mrd.22130 23109270 PMC3923310

[JEB247775C25] Lahnsteiner, F. (2014). The effect of K^+^, Ca^2+^, and Mg^2+^ on sperm motility in the perch, *Perca fluviatilis*. *Fish Physiol. Biochem.* 40, 469-480. 10.1007/s10695-013-9858-7 24037272

[JEB247775C26] Lakowicz, J. R., Szmacinski, H., Nowaczyk, K. and Johnson, M. L. (1992). Fluorescence lifetime imaging of free and protein-bound NADH. *Proc. Natl. Acad. Sci. U.S.A.* 89, 1271-1275. 10.1073/pnas.89.4.1271 1741380 PMC48431

[JEB247775C27] Machinist, J. M. and Singer, T. P. (1965). Reactions of coenzyme Q in the DPNH dehydrogenase segment of the respiratory chain. *Proc. Natl. Acad. Sci. U.S.A.* 53, 467-474. 10.1073/pnas.53.2.467 14294083 PMC219536

[JEB247775C28] Massino, C., Wetzker, C., Kremenova, J., Sasinkova, M., Balvin, O., Bartonicka, T., Otti, O. and Reinhardt, K. (2022). Seminal fluid and sperm diluent affect sperm metabolism in an insect: evidence from NAD(P)H and flavin adenine dinucleotide autofluorescence lifetime imaging. *Microsc Res. Techn.* 85, 398-411. 10.1002/jemt.23914 34486193

[JEB247775C29] McComb, R. B. and Yushok, W. D. (1964). Metabolism of ascites tumor cells. III. Effect of 2-deoxyglucose phosphorylation on phosphorus metabolism. *Cancer Res.* 24, 193-197. 14115683

[JEB247775C30] Meleshina, A. V., Dudenkova, V. V., Bystrova, A. S., Kuznetsova, D. S., Shirmanova, M. V. and Zagaynova, E. V. (2017). Two-photon FLIM of NAD(P)H and FAD in mesenchymal stem cells undergoing either osteogenic or chondrogenic differentiation. *Stem Cell Res. Ther.* 8, 15. 10.1186/s13287-017-0484-7 28129796 PMC5273806

[JEB247775C31] Moraes, C. R. and Meyers, S. (2018). The sperm mitochondrion: organelle of many functions. *Anim. Reprod. Sci.* 194, 71-80. 10.1016/j.anireprosci.2018.03.024 29605167

[JEB247775C32] Nakashima, N., Yoshihara, K., Tanaka, F. and Yagi, K. (1980). Picosecond fluorescence lifetime of the coenzyme of D-amino acid oxidase. *J. Biol. Chem.* 255, 5261-5263. 10.1016/S0021-9258(19)70779-0 6102996

[JEB247775C33] Orr, T. J. and Brennan, P. L. R. (2015). Sperm storage: distinguishing selective processes and evaluating criteria. *Trends Ecol. Evol.* 30, 261-272. 10.1016/j.tree.2015.03.006 25843274

[JEB247775C34] Paynter, E., Millar, A. H., Welch, M., Baer-Imhoof, B., Cao, D. and Baer, B. (2017). Insights into the molecular basis of long-term storage and survival of sperm in the honeybee (*Apis mellifera*). *Sci. Rep.* 7, 40236. 10.1038/srep40236 28091518 PMC5238380

[JEB247775C35] Piomboni, P., Focarelli, R., Stendardi, A., Ferramosca, A. and Zara, V. (2012). The role of mitochondria in energy production for human sperm motility. *Int. J. Androl.* 35, 109-124. 10.1111/j.1365-2605.2011.01218.x 21950496

[JEB247775C36] Pitnick, S., Wolfner, M. F. and Dorus, S. (2020). Post-ejaculatory modifications to sperm (PEMS). *Biol. Rev.* 95, 365-392. 10.1111/brv.12569 31737992 PMC7643048

[JEB247775C37] Poiani, A. (2006). Complexity of seminal fluid: a review. *Behav. Ecol. Sociobiol.* 60, 289-310. 10.1007/s00265-006-0178-0

[JEB247775C38] Prokupek, A. M., Kachman, S. D., Ladunga, I. and Harshman, L. G. (2009). Transcriptional profiling of the sperm storage organs of *Drosophila melanogaster*. *Insect Mol. Biol.* 18, 465-475. 10.1111/j.1365-2583.2009.00887.x 19453766

[JEB247775C39] Rangel, J., Shepherd, T. F., Gonzalez, A. N., Hillhouse, A., Konganti, K. and Ing, N. H. (2021). Transcriptomic analysis of the honey bee (*Apis mellifera*) queen spermathecae reveals genes that may be involved in sperm storage after mating. *PloS ONE* 16, e0244648. 10.1371/journal.pone.0244648 33417615 PMC7793254

[JEB247775C40] Reinhardt, K. (2005). Sperm numbers, sperm storage duration and fertility limitation in the Odonata. *Int. J. Odonatol.* 8, 45-58. 10.1080/13887890.2005.9748242

[JEB247775C41] Reinhardt, K. (2007). Evolutionary consequences of sperm cell aging. *Q. Rev. Biol.* 82, 375-393. 10.1086/522811 18217528

[JEB247775C42] Reinhardt, K. and Ribou, A.-C. (2013). Females become infertile as the stored sperm's oxygen radicals increase. *Sci. Rep.* 3, 02888. 10.1038/srep02888

[JEB247775C43] Reinhardt, K., Dobler, R. and Abbott, J. (2015). An ecology of sperm: sperm diversification by natural selection. *A. Rev. Ecol., Evol. Syst.* 46, 435-459. 10.1146/annurev-ecolsys-120213-091611

[JEB247775C44] Reinhardt, K., Kohler, G. and Schumacher, J. (1999). Females of the grasshopper *Chorthippus parallelus* (Zett.) do not remate for fresh sperm. *Proc. R.Soc. Biol. Sci.* 266, 2003-2009. 10.1098/rspb.1999.0878

[JEB247775C45] Ribou, A.-C. and Reinhardt, K. (2012). Reduced metabolic rate and oxygen radicals production in stored insect sperm. *Proc. R.Soc. Biol. Sci.* 279, 2196-2203. 10.1098/rspb.2011.2422 PMC3321705 22279170

[JEB247775C46] Ridley, M. (1988). Mating frequency and fecundity in insects. *Biol. Rev.* 63, 509-549. 10.1111/j.1469-185X.1988.tb00669.x

[JEB247775C47] Sarkans, U., Gostev, M., Athar, A., Behrangi, E., Melnichuk, O., Ali, A., Minguet, J., Rada, J. C., Snow, C., Tikhonov, A. et al. (2018). The BioStudies database-one stop shop for all data supporting a life sciences study. *Nucleic Acids Res.* 46, D1266-D1270. 10.1093/nar/gkx965 29069414 PMC5753238

[JEB247775C48] Schnakenberg, S. L., Matias, W. R. and Siegal, M. L. (2011). Sperm-storage defects and live birth in Drosophila females lacking spermathecal secretory cells. *PLoS Biol.* 9, e1001192. 10.1371/journal.pbio.1001192 22087073 PMC3210755

[JEB247775C49] Skala, M. C., Riching, K. M., Gendron-Fitzpatrick, A., Eickhoff, J., Eliceiri, K. W., White, J. G. and Ramanujam, N. (2007). *In vivo* multiphoton microscopy of NADH and FAD redox states, fluorescence lifetimes, and cellular morphology in precancerous epithelia. *Proc. Natl. Acad. Sci. U.S.A.* 104, 19494-19499. 10.1073/pnas.0708425104 18042710 PMC2148317

[JEB247775C50] Stringari, C., Nourse, J. L., Flanagan, L. A. and Gratton, E. (2012). Phasor fluorescence lifetime microscopy of free and protein-bound NADH reveals neural stem cell differentiation potential. *PloS ONE* 7, e48014. 10.1371/journal.pone.0048014 23144844 PMC3489895

[JEB247775C51] Tan, C. K. W., Pizzari, T. and Wigby, S. (2013). Parental age, gametic age, and inbreeding interact to modulate offspring viability in *Drosophila melanogaster*. *Evolution* 67, 3043-3051. 24094353 10.1111/evo.12131

[JEB247775C52] Tourmente, M., Villar-Moya, P., Rial, E. and Roldan, E. R. S. (2015). Differences in ATP generation via glycolysis and oxidative phosphorylation and relationships with sperm motility in mouse species. *J. Biol. Chem.* 290, 20613-20626. 10.1074/jbc.M115.664813 26048989 PMC4536464

[JEB247775C53] Turnell, B. R. and Reinhardt, K. (2020). Metabolic rate and oxygen radical levels increase but radical generation rate decreases with male age in *Drosophila melanogaster* sperm. *J. Gerontol A* 75, 2278-2285. 10.1093/gerona/glaa078 32267495

[JEB247775C54] Turnell, B. R. and Reinhardt, K. (2022). Sperm metabolic rate predicts female mating frequency across *Drosophila* species. *Evolution* 76, 573-584. 10.1111/evo.14435 35064568

[JEB247775C55] Turnell, B. R., Kumpitsch, L., Ribou, A.-C. and Reinhardt, K. (2021a). Somatic production of reactive oxygen species does not predict its production in sperm cells across *Drosophila melanogaster* lines. *BMC Res. Notes* 14, 131. 10.1186/s13104-021-05550-7 33827685 PMC8028716

[JEB247775C56] Turnell, B. R., Kumpitsch, L. and Reinhardt, K. (2021b). Production and scavenging of reactive oxygen species both affect reproductive success in male and female *Drosophila melanogaster*. *Biogerontology* 22, 379-396. 10.1007/s10522-021-09922-1 33903991 PMC8266701

[JEB247775C57] van der Velden, E. (2020). CMasher: Scientific colormaps for making accessible, informative and “cmashing” plots. *J. Open Source Softw* 5, 2004. 10.21105/joss.02004

[JEB247775C58] van Rossum, G. (1995). Python tutorial. Technical Report CS-R9526. Amsterdam: Centrum voor Wiskunde en Informatica (CWI).

[JEB247775C59] Wetzker, C. and Reinhardt, K. (2019). Distinct metabolic profiles in Drosophila sperm and somatic tissues revealed by two-photon NAD(P)H and FAD autofluorescence lifetime imaging. *Sci. Rep.* 9, 19534. 10.1038/s41598-019-56067-w 31862926 PMC6925207

[JEB247775C60] Wigby, S., Brown, N. C., Allen, S. E., Misra, S., Sitnik, J. L., Sepil, I., Clark, A. G. and Wolfner, M. F. (2020). The *Drosophila* seminal proteome and its role in postcopulatory sexual selection. *Philos. Trans. R. Soc. Lond. B Biol. Sci.* 375, 20200072. 10.1098/rstb.2020.0072 33070726 PMC7661438

[JEB247775C61] Wobbrock, J. O., Findlater, L., Gergle, D. and Higgins, J. J. (2011). The aligned rank transform for nonparametric factorial analyses using only anova procedures. Proceedings of the SIGCHI Conference on Human Factors in Computing Systems. CHI ‘11: CHI Conference on Human Factors in Computing Systems, New York, NY, USA: ACM.

[JEB247775C62] Woodward, G. E. and Cramer, F. B. (1952). 2-Desoxyl-D-glucose as an inhibitor of anaerobic glycolysis in tumor tissue. *J. Franklin Institute* 254, 259-260. 10.1016/0016-0032(52)90482-1

