# Supplementary material for: Drosophila melanogaster sperm turn more oxidative in the female
Source: J Exp Biol. 2024 Aug 7;227(15):jeb247775. doi: 10.1242/jeb.247775 (PMC11418169; doi:10.1242/jeb.247775)
Supplement: Supplementary information [file jexbio-227-247775-s1.pdf]

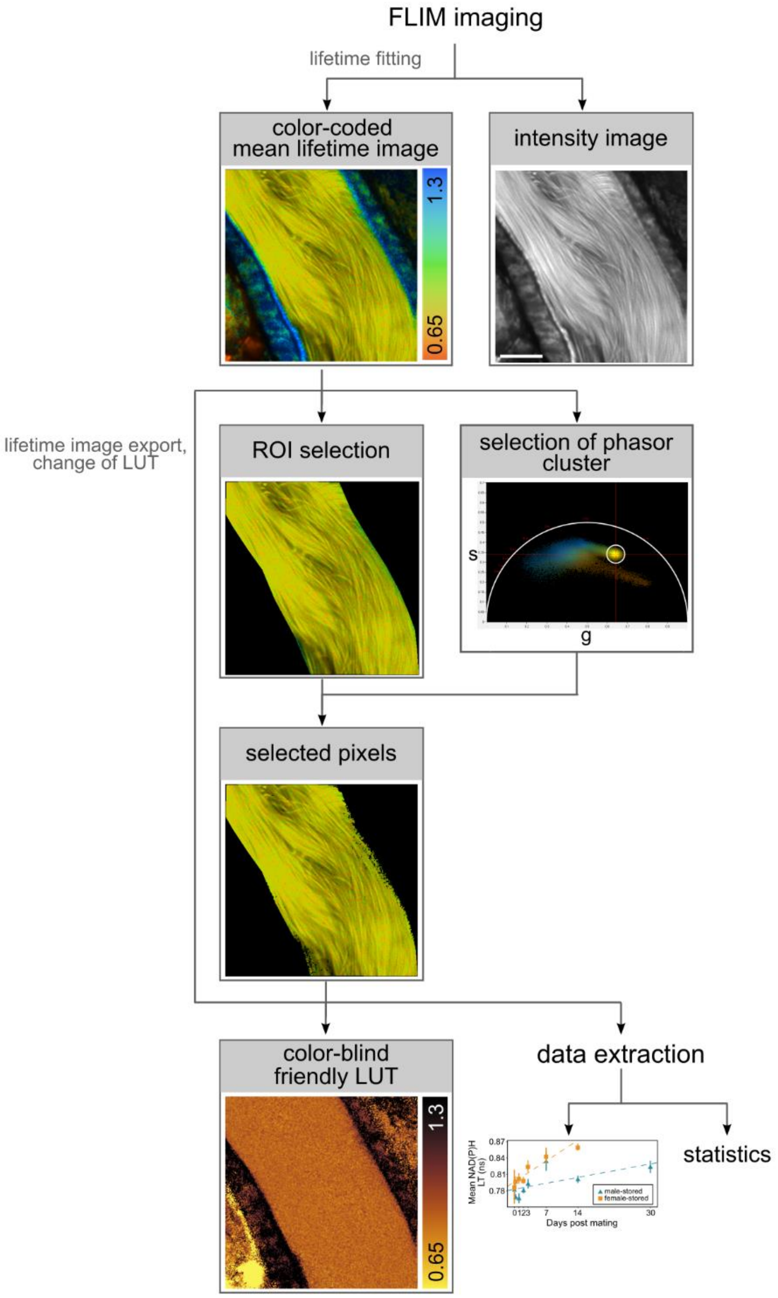

Fig. S1. Image processing workflow. Spatial and lifetime-based selection of pixels for downstream lifetime analysis. Scale bar is 10 µm. To extract sperm-specific FLIM parameters from images, we applied the following workflow. Firstly, regions of interest with sperm were manually selected in color-coded lifetime images with careful exclusion of epithelial structures. The created image mask shows selected pixel in color and excluded pixels in black. Secondly, structures with clearly different lifetime patterns, normally being signal from epithelia visible in regions of low sperm density, were further excluded from analysis based on selection of clusters from the corresponding phasor plot. Pixels after spatial and phasor-based lifetime selection were used for further quantitative lifetime analysis.

Journal of Experimental Biology • Supplementary information

Table S1. Sample sizes of male and female flies by storage duration for NAD(P)H and FAD FLIM measurements. Numbers of high quality samples used for analysis/ number of samples imaged based on image quality thresholds. Groups excluded from plots due to low sample size are indicated with an asterisk.

Time post mating (days)	Location: Male		Location: Female	
	NAD(P)H	FAD	NAD(P)H	FAD
0	7/10	7/7	5/10	5/8
0.25	4/7	3/4	4/7	1*/4
1	5/9	4/6	6/9	6/7
2	6/7	4/6	7/8	5/8
3	6/7	7/7	7/7	6/7
7	5/5	5/5	6/6	6/6
14	8/8	6/6	3/9	7/7
30	7/9	4/6	0*/0	0*/0

Table S2. The effect of chemical inhibition (treatment) and sperm density on sperm metabolism in *D. melanogaster in vitro*. Sperm density in FLIM images did not significantly affect either the mean NAD(P)H lifetime (A) or the short NAD(P)H lifetime fraction (B) in the *in vitro* analyses. Note that extremely low sperm densities (*) were excluded from the analysis.

A	τ_m	Df	Sum of squares	Mean Squares	F value	Pr(>F)
	Treatment	2	3639256	1819678	7.4723	0.0038**
	Density	1	36321	36321	0.1491	0.7034
	Treatment: Density	2	1069122	534561	2.1951	0.1374
	Residuals	20	48705456	243523		

B	a_1%	Df	Sum of squares	Mean Squares	F value	Pr(>F)
	Treatment	2	841.82	420.91	7.1150	0.0046**
	Density	1	2.09	2.09	0.0353	0.8528
	Treatment: Density	2	32.14	16.07	0.2717	0.7649
	Residuals	20	1183.16	59.16		

Lifetime parameters are not homogeneous across images of sperm *in vitro* possibly due to variations in sperm density. We, therefore, asked one observer blind to treatment to score sperm density on a scale from 1 to 4 (very high to very low). We found no general pattern for sperm density 1 to 3 but very low sperm density was associated with the most extreme a_1% values. We therefore excluded very low density data and re-ran our analyses with sperm density as a covariate. Sperm density had not significant effect, nor had its interaction with treatment.
